# K_Ca_3.1 channel blockade attenuates microvascular remodelling in a large animal model of bleomycin-induced pulmonary fibrosis

**DOI:** 10.1038/s41598-019-56412-z

**Published:** 2019-12-27

**Authors:** Habtamu B. Derseh, Sasika N. Vithana Dewage, Kopiyawaththage U. E. Perera, Charles N. Pagel, Emmanuel Koumoundouros, Louise Organ, Ken J. Snibson

**Affiliations:** 1 Faculty of Veterinary and Agricultural Sciences, The University of Melbourne, Parkville, Victoria Australia; 2 Department of Electrical and Electronic Engineering, The University of Melbourne, Parkville, Victoria Australia; 3 Division of Respiratory Medicine, University of Nottingham, Nottingham, UK

**Keywords:** Imaging, Mechanisms of disease, Respiratory tract diseases

## Abstract

Idiopathic pulmonary fibrosis (IPF) is a chronic progressive lung disease with limited therapeutic options and poor prognosis. IPF has been associated with aberrant vascular remodelling, however the role of vascular remodelling in pulmonary fibrosis is poorly understood. Here, we used a novel segmental challenge model of bleomycin-induced pulmonary fibrosis in sheep to evaluate the remodelling of the pulmonary vasculature, and to investigate the changes to this remodelling after the administration of the K_Ca_3.1 channel inhibitor, senicapoc, compared to the FDA-approved drug pirfenidone. We demonstrate that in vehicle-treated sheep, bleomycin-infused lung segments had significantly higher blood vessel density when compared to saline-infused control segments in the same sheep. These microvascular density changes were significantly attenuated by senicapoc treatment. The increases in vascular endothelial growth factor (VEGF) expression and endothelial cell proliferation in bleomycin-infused lung segments were significantly reduced in sheep treated with the senicapoc, when compared to vehicle-treated controls. These parameters were not significantly suppressed with pirfenidone treatment. Senicapoc treatment attenuated vascular remodelling through inhibition of capillary endothelial cell proliferation and VEGF expression. These findings suggest a potential new mode of action for the novel drug senicapoc which may contribute to its efficacy in combatting pulmonary fibrosis.

## Introduction

Idiopathic pulmonary fibrosis (IPF) is a chronic progressive lung disease of unknown aetiology with limited therapeutic options and extremely poor prognosis. It is characterised by deposition of excess extracellular matrix within the lung interstitium and remodelling of lung parenchyma. The pathogenesis of IPF is poorly understood, though current paradigms focus on alveolar epithelial cell injury as a key initiating event followed by dysregulated wound healing process resulting in fibrosis and distortion of the lung’s architecture. During this process, injuries to the lung not only damage the normal lung parenchyma but also affects the pulmonary vasculature leading to an aberrant vascular remodelling^1^. The presence of aberrant vascular remodelling in IPF was first described by Turner-Warwick^2^, who demonstrated extensive neovascularization in the areas of fibrosis and anastomosis between pulmonary and systemic microvasculature in lung specimens from patients with IPF. While other studies have confirmed that vascular remodelling plays role in the development of pulmonary fibrosis^3–7^, the mechanisms that operate in the disease processes are largely unexplored.

Vascular remodelling plays key roles in normal wound healing processes and its modulation may also be important in regulating development of fibrotic disorders. Therefore, a better understanding of the contribution of aberrant vascular remodelling to the pathogenesis of pulmonary fibrosis is crucial to develop novel therapeutic agents. Importantly, in rat and mouse models of bleomycin-induced pulmonary fibrosis, pulmonary fibrosis is also attenuated if vascular remodelling is specifically impeded^3–5,8,9^. Furthermore, in these studies a number of different experimental settings have been used to impede *in vivo* angiogenesis to examine its effects on fibrosis, including: the systemic administration of IFN-gamma-inducible protein-10^3,4^; neutralization of the angiogenic CXC chemokine-macrophage inflammatory protein-2^5^; treatment with the chemokine receptor CXCR2 antagonist^8^; and administration of the angiogenesis inhibitor, endostatin^9^. In all these studies, a retardation of angiogenesis resulted in reduced bleomycin-induced pulmonary fibrosis.

Currently, no pharmacological treatment is able to cure IPF and other treatment options are very limited. While pirfenidone and nintedanib have been licensed for the treatment of IPF and have been shown to slow down the progression of the disease and decline in lung function^10–12^, these drugs have been associated with unwanted gastrointestinal and skin-related adverse effects that can result in discontinuation of medication in some patients^13^. We have recently developed a physiologically and pharmacologically relevant large animal model for pulmonary fibrosis which allows for the exploration of the disease mechanisms, as well as for the investigation of new treatments for this disease^14^. In this model, fibrotic remodelling and correspondingly poor lung function has been shown to persist for at least seven weeks after bleomycin injury^15,16^ making it a useful preclinical model to investigate novel anti-fibrotic therapeutic agents.

In the current study, we used the sheep model of pulmonary fibrosis to test the effects of a novel drug senicapoc on vascular remodelling in pulmonary fibrosis. This drug inhibits the intermediate-conductance calcium-activated K^+^ channel (K_Ca_3.1) (also known as IK1, SK4, or KCNN4). This ion channel regulates membrane potential and calcium signalling in many cell types including those integral to the pathogenesis of IPF such as fibroblasts, epithelial cells and endothelial cells^17,18^. Recently we have shown that selective inhibition of K_Ca_3.1 ion channel alleviates established fibrosis as well as improves lung function in sheep lung segments exposed to bleomycin-induced fibrosis^16^. This study evaluates the presence of vascular remodelling during the development of bleomycin-induced pulmonary fibrosis and investigates the effects senicapoc on vascular remodelling when compared to the FDA-approved drug pirfenidone. Some of the results of these studies have been previously reported in the form of an abstract^19^.

## Results

### Vascular remodelling in a large animal model of bleomycin-induced pulmonary fibrosis

**Figure 1:**
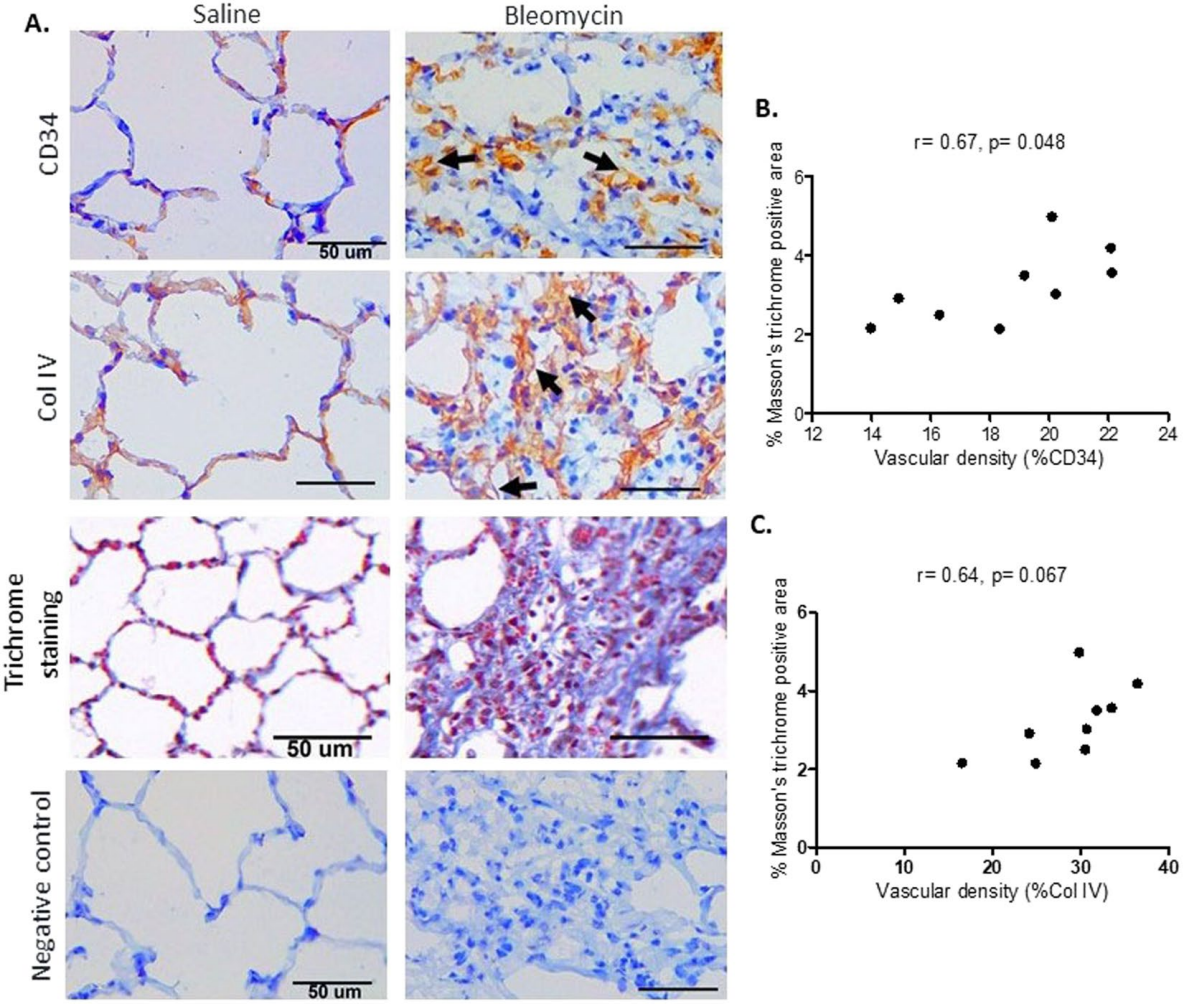
Alveolar capillaries in saline and bleomycin treated lung segments. (**A**) The panels show representative histological sections of either saline-infused (left-side images), or bleomycin-infused (right-side images), lung segments taken from the same sheep. The top two panels show sections that were immuno-stained with antibodies against either CD34 or collagen type IV to detect capillaries in parenchyma. Arrows show large and dilated capillaries in bleomycin treated segment. The third panel shows representative Masson’s trichrome-stained sections that were used to determine fibrotic fraction. Primary antibodies were omitted in the negative control (fourth panel). Graphs show the relationship between vascular density and fibrotic fraction in bleomycin infused lung segments immuno-stained with CD34 (*r* = 0.67, *p* = 0.048) (**B**) and collagen type IV (*r* = 0.64, *p* = 0.067) (**C**) (n = 9 sheep). Scale bars = 50 µm.

To elucidate the presence of vascular remodelling in the parenchyma of lung during the development of bleomycin-induced pulmonary fibrosis in sheep, we used antibodies against CD34 and collagen type IV on frozen lung sections to identify different structures in blood vessels. Whilst collagen type IV stains the basement membrane of blood vessels, CD34 stains transmembrane glycoprotein in endothelial cells. In bleomycin infused lung segments immunoreactivity to both markers of blood vessels were increased compared to saline infused internal control lung segments indicating angiogenesis (neoformation of blood vessels) induced by bleomycin administration (Fig. 1A). In addition, in bleomycin treated lung segments capillaries were large and dilated. In both control and fibrotic lung segments, collagen type IV stained more blood vessels compared to CD34 (Fig. 1A).

Correlation analysis was performed to evaluate the relationship of observed vascularity in the parenchyma of fibrotic lungs with collagen deposition. Interestingly we found positive correlation between increased vascular density using both blood vessel markers and collagen deposition in bleomycin infused lung segments. There was significant positive correlation between vascular density of CD34 stained capillaries and fibrotic fraction in bleomycin infused lobes (*r* = 0.67, *p* = 0.048) (Fig. 1B). However, the correlations between increased density of collagen type IV immuno-stained blood vessels with fibrotic fraction did not reach significance (*r* = 0.64, *p* = 0.067). We found no significant correlation between increased vascular density and lung function changes (data not shown).

### Effects of senicapoc and pirfenidone on vascular remodelling

**Figure 2:**
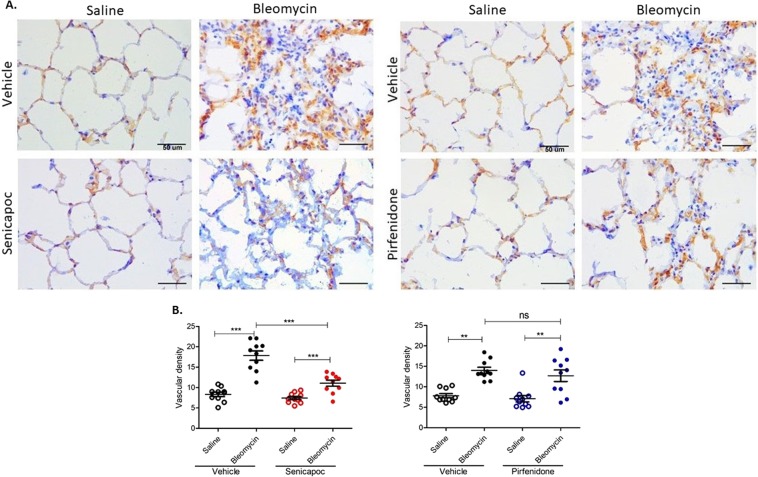
Effects of senicapoc and pirfenidone on remodelling of CD34 stained capillaries induced by bleomycin. (**A**) The panels show representative anti-CD34 immuno-stained sections of lung segments from sheep that were treated with either vehicle, senicapoc or pirfenidone. The lung segments from individual sheep were infused with either saline (left-side images), or bleomycin (right-side images). (**B**) Shows the vascular density data between the different treatment groups. Vascular density was determined by the percentage of the total area of CD34-positive vascular area per field of lung parenchyma. Ten images were taken from fibroproliferative areas for each lobe from each sheep (n = 10) and the values for each of the ten images were averaged for each lobe. Data show individual values plus mean. Significance was determined using a paired *t* test between lobes from the same treatment group, and an unpaired *t* test between lobes from different groups, **p < 0.01, ***p < 0.001, ns-not significant. Data represent mean (±SEM). Scale bars = 50 µm.

**Figure 3:**
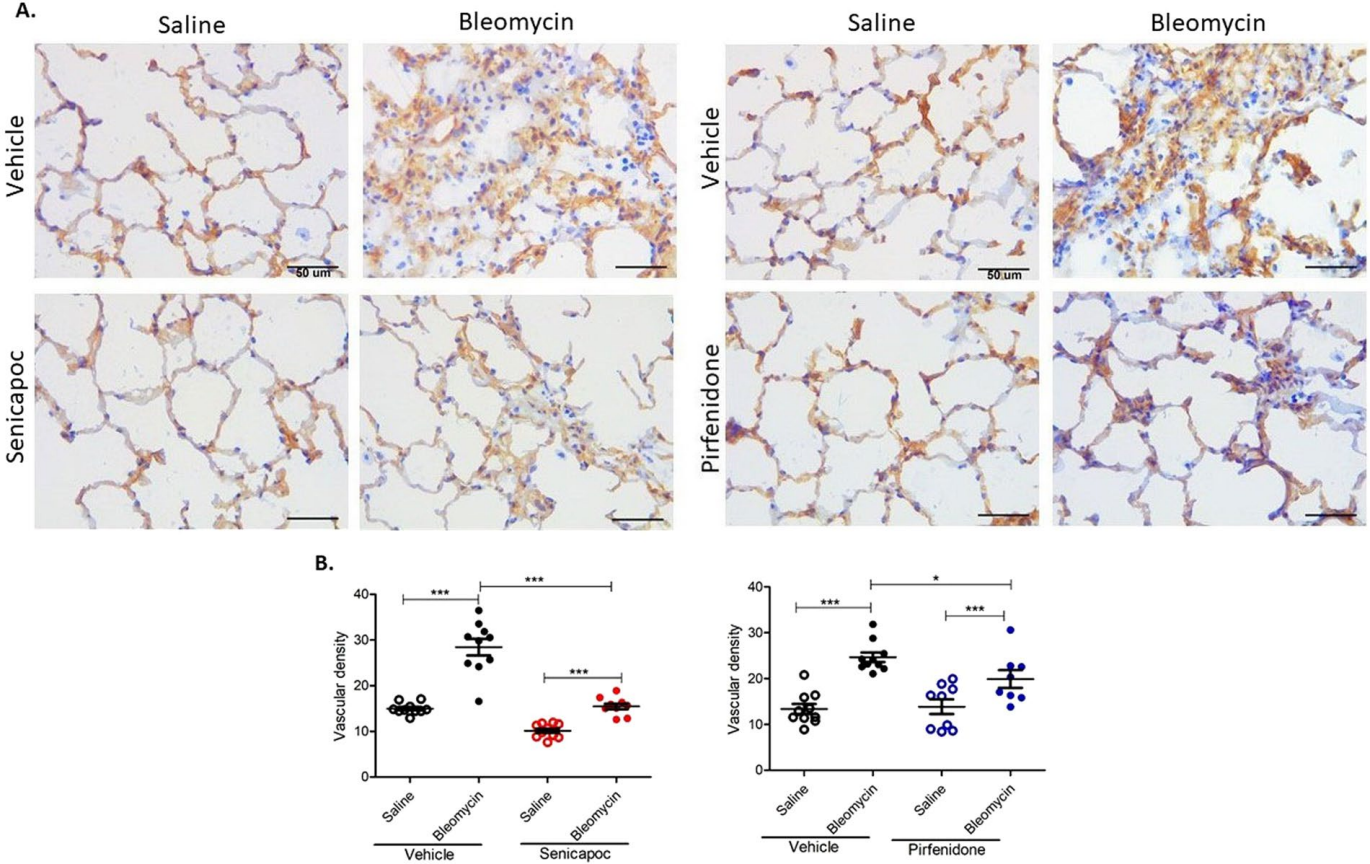
Effects of senicapoc and pirfenidone on remodelling of collagen type IV stained capillaries induced by bleomycin. (**A**) The panels show representative anti-collagen type IV immuno-stained sections of lung segments from sheep that were treated with either vehicle, senicapoc or pirfenidone. The lung segments from individual sheep were infused with either saline (left-side images), or bleomycin (right-side images). (**B**) Shows the vascular density data between the different treatment groups. Vascular density was determined by the percentage of the total area of anti-collagen type IV-positive vascular area per field of lung parenchyma. Ten images were taken from fibroproliferative areas for each lobe from each sheep (n = 10) and the values for each of the ten images were averaged for each lobe. Data show individual values plus mean. Significance was determined using a paired *t* test between lobes from the same treatment group and an unpaired *t* test between lobes from different groups, *p < 0.05, ***p < 0.001. Data represent mean (±SEM). Scale bars = 50 µm.

As shown in Figs. 2A and 3A, there was prominent fibrosis in bleomycin infused segments compared to saline infused segments in sheep without drug intervention. This was noticeably reduced in sheep treated with senicapoc and pirfenidone. In sheep without drug intervention, there was significant increased density of both CD34-positive (Fig. 2B vehicle) and collagen type IV- positive (Fig. 3B vehicle) capillaries in the parenchyma of bleomycin infused segments compared to control segments indicating neovascularization induced by bleomycin administration. This augmentation of blood vessel density was significantly reduced by senicapoc treatment (Figs. 2B and 3B). Senicapoc reduced both CD34 and collagen type IV immuno-stained capillaries (CD34-immunopositivity: bleomycin-vehicle 17.84 ± 1.14% vs bleomycin-senicapoc 11.08 ± 0.74%, *p* < 0.0001, and collagen type IV-immunopositivity: bleomycin-vehicle 28.44 ± 1.8% vs bleomycin-senicapoc 15.5 ± 0.6%, *p* < 0.0001) (Figs. 2B and 3B). When we compare saline-infused lobes in senicapoc and vehicle-treated animals, systemic senicapoc treatment via the oral route has a small effect on the saline-infused lung segment, as well as its obvious effect on the bleomycin-infused lung segment in the contralateral lung lobe for collagen type IV stained capillaries only (Fig. 3B). On the other hand, pirfenidone significantly attenuated vascular remodelling of collagen type IV stained capillaries but not CD34 stained capillaries (Figs. 2B and 3B). The administration of pirfenidone reduced the density of blood vessels detected by type IV collagen staining in bleomycin-pirfenidone treated segments when compared to bleomycin- vehicle treated segments (24.67 ± 1.04% (bleomycin-vehicle), vs 19.93 ± 1.91, (bleomycin-pirfenidone) *p* = 0.0353) (Fig. 3B). However, pirfenidone did not significantly decrease density of CD34 positive capillaries (Fig. 2B). The group comparison between senicapoc- and pirfenidone-treated sheep in bleomycin-injured segments shows a significant lower mean vascular density in senicapoc-treated animals for collagen IV stained capillaries (15.5 ± 0.6 bleomycin-senicapoc vs 19.93 ± 1.91, (bleomycin-pirfenidone, p = 0.0271), but not for CD34 stained capillaries (bleomycin-senicapoc 11.08 ± 0.74%, vs bleomycin-pirfenidone 12.7 ± 1.4%, p = 0.3179).

### Senicapoc, but not pirfenidone, reduces VEGF expression

**Figure 4:**
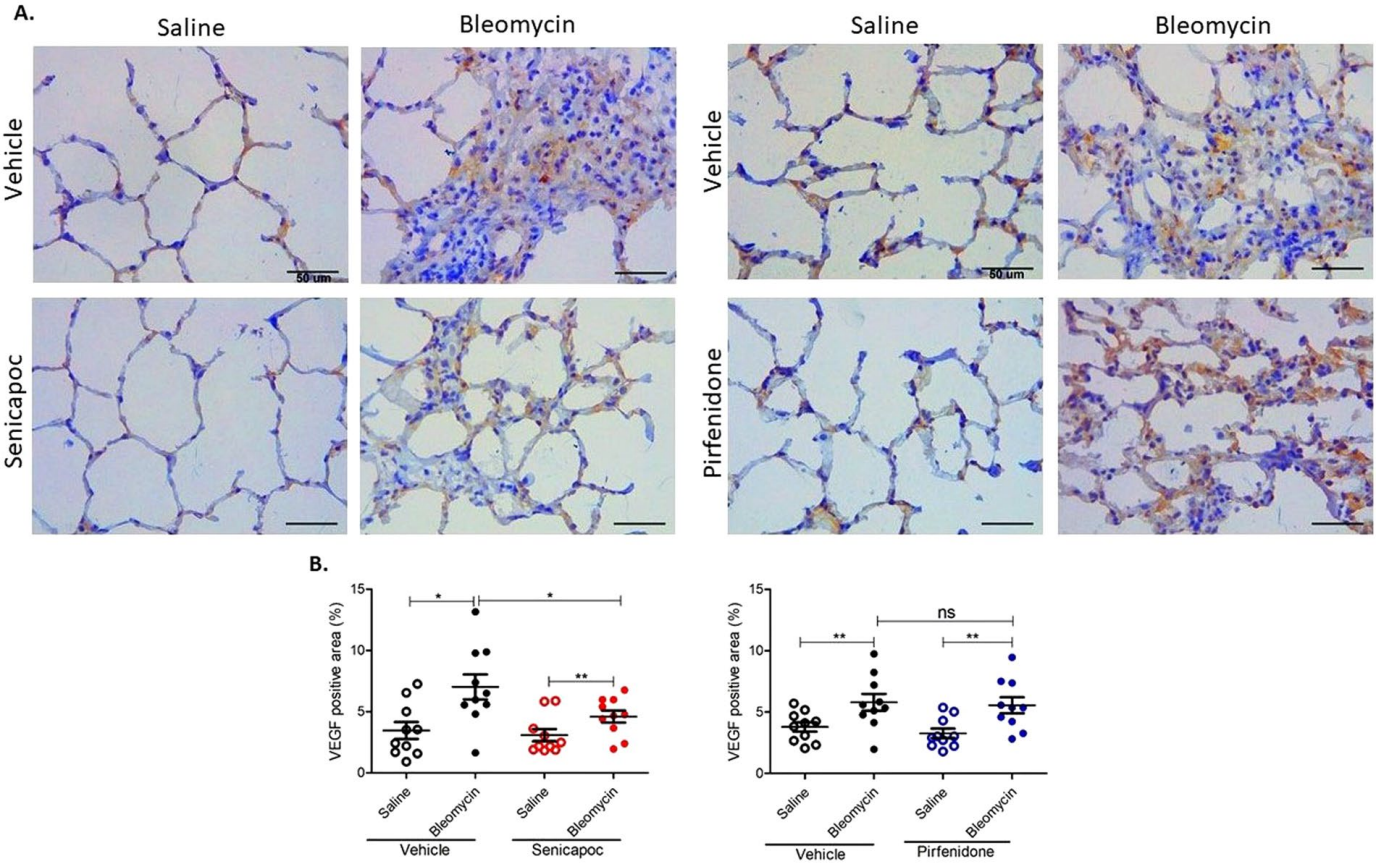
Senicapoc, but not pirfenidone, down regulates VEGF expression that was increased by bleomycin. (**A**) The panels show representative anti-VEGFA immuno-stained sections of lung segments from sheep that were treated with either vehicle, senicapoc or pirfenidone. The lung segments from individual sheep were infused with either saline (left-side images), or bleomycin (right-side images). (**B**) Shows the data for the percentage VEGF-positive area per total field area of lung parenchyma for the different treatment groups. Data show individual values plus mean, (n = 10 sheep). Significance was determined using a paired *t* test between lobes from the same treatment group and an unpaired *t* test between lobes from different groups, *p < 0.05, **p < 0.01, ns-not significant. Data represent mean (±SEM). Scale bars = 50 µm.

VEGF expression was visibly higher in bleomycin infused lung segments compared to saline infused control segments in vehicle treated animals (Fig. 4A). However, there was less abundant expression of VEGF in bleomycin infused segments of senicapoc treated sheep but not in pirfenidone treated sheep as shown in Fig. 4A. Analysis of the percentage of the VEGF positive area per total field of lung parenchyma (Fig. 4B), revealed that there was significantly higher VEGF positive area in bleomycin infused lung segments compared to saline infused segments in sheep without drug intervention (% VEGF positive area/total field: saline-vehicle 3.41 ± 0.68 vs bleomycin-vehicle 7.03 ± 1.02 p = 0.0177 for senicapoc study; saline-vehicle 3.79 ± 0.39 vs bleomycin-vehicle 5.8 ± 0.69, *p* = 0.0041 for pirfenidone study). This increased VEGF expression in fibrotic lung segments was significantly reduced by senicapoc treatment (% VEGF positive area/total field: bleomycin-vehicle 7.03 ± 1.02 vs bleomycin-senicapoc 4.6 ± 0.5, *p* = 0.0454) but not by pirfenidone treatment (% VEGF positive area/total field: bleomycin-vehicle 5.8 ± 0.69 vs bleomycin-pirfenidone 5.56 ± 0.65, p = 0.8015, Fig. 4B). The group comparison between senicapoc- and pirfenidone-treated sheep in bleomycin-injured segments shows that VEGF expression was lower with senicapoc treatment, but not significantly different to pirfenidone treatment, (% VEGF positive area/total field: bleomycin-senicapoc 4.6 ± 0.5 vs bleomycin-pirfenidone 5.56 ± 0.65, p = 0.2557).

### K_ca_3.1 channel inhibition, but not pirfenidone treatment, attenuates bleomycin-induced proliferation of alveolar capillary endothelial cells *in vivo*

**Figure 5:**
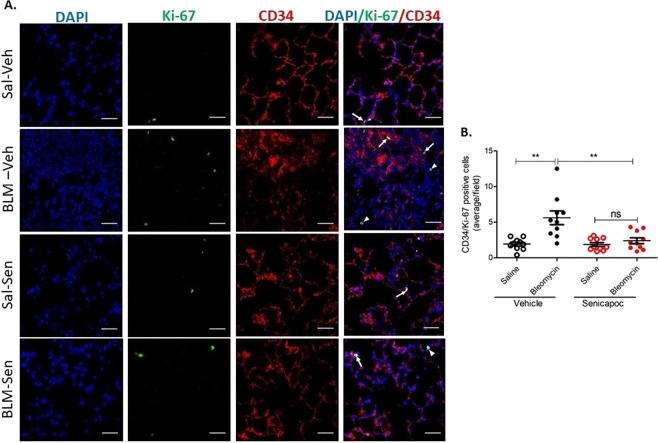
Senicapoc reduces alveolar capillary endothelial proliferation *in vivo*. (**A**) Representative immunofluorescence photomicrograph of lung cryosections from differentially treated lung segments double stained with Ki-67 and CD34 antibodies. CD34 stained vessels together with Ki-67 signals indicate endothelial cell proliferation (arrows indicate Ki-67 overlapping with CD34 immunofluuorescence). Arrowheads indicate proliferating non-endothelial cells. (**B**) Shows data for CD34/Ki-67 positive cells from the different treatment groups (average/10 fields in each differentially treated segments, n = 10 sheep), **p < 0.01, ns-not significant. Data represent mean (±SEM). Sal-Veh = saline-vehicle lobe; BLM-Veh = bleomycin-vehicle lobe; Sal-Sen = saline-senicapoc lobe; BLM-Sen = bleomycin-senicapoc lobe. Scale bars = 50 µm.

**Figure 6:**
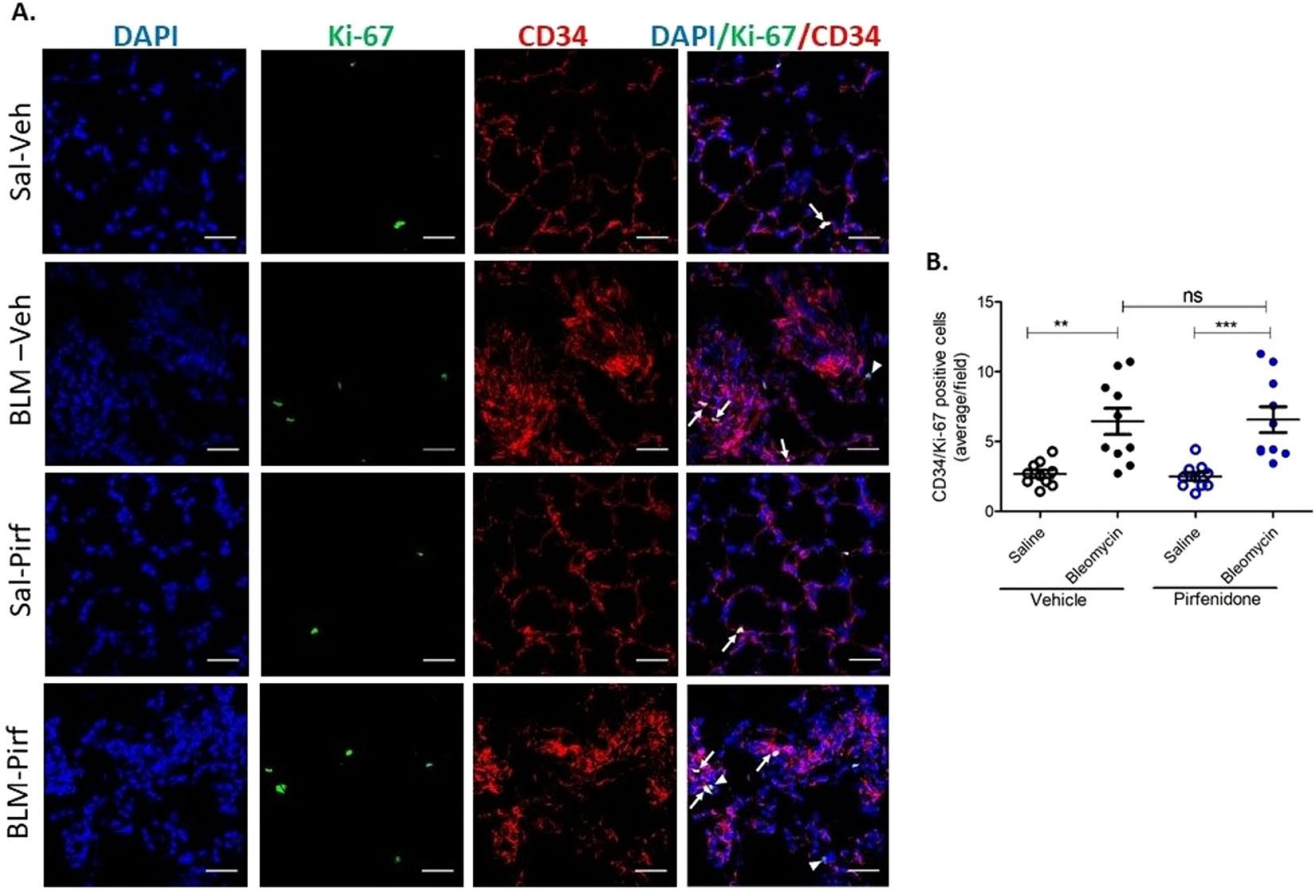
Pirfenidone does not reduce alveolar capillary endothelial proliferation *in vivo*. (**A**) Representative immunofluorescence photomicrograph of lung cryosections from differentially treated lung segments double stained with Ki-67 and CD34 antibodies. CD34 stained vessels together with Ki-67 signals indicate endothelial cell proliferation (arrows indicate Ki-67 overlapping with CD34 immunofluuorescence). Arrowheads indicate proliferating non-endothelial cells. (**B**) Shows data for CD34/Ki-67 positive cells from the different treatment groups (average/10 fields in each differentially treated segments, n = 10 sheep), **p < 0.01, ***p < 0.001, ns-not significant. Data represent mean (±SEM). Sal-Veh = saline-vehicle lobe; BLM-Veh = bleomycin-vehicle lobe; Sal-Pirf = saline-pirfenidone lobe; BLM-Pirf = bleomycin-pirfenidone lobe. Scale bars = 50 µm.

Inhibition of the K_Ca_3.1 channel has been previously shown to have anti-angiogenic effects^18^. Because senicapoc suppressed angiogenesis in bleomycin-induced pulmonary fibrosis, we asked whether senicapoc would reduce endothelial proliferation *in vivo* in the fibrotic lung segments in sheep. To this end, we double-labelled the lung section of each differentially treated segments with Ki-67 (proliferation marker) and CD34 (endothelial marker) specific antibodies and evaluated proliferation of capillary endothelial cells. As expected endothelial proliferation was less in saline-vehicle treated lobes (Figs. 5A and 6A). In contrast, more frequent proliferating endothelial cells were observed in bleomycin-vehicle treated lobes (CD34/Ki-67 positive cells, average/field: saline-vehicle 1.91 ± 0.26 vs bleomycin-vehicle 5.6 ± 0.96, *p* = 0.0013 for senicapoc study; saline-vehicle 2.69 ± 0.27 vs bleomycin-vehicle 6.44 ± 0.94, *p* = 0.0017 for pirfenidone study). Importantly, administration of senicapoc, but not pirfenidone treatment, significantly reduced proliferation of endothelial cells in bleomycin infused lung segments (CD34/Ki-67 positive cells, average/field: bleomycin-vehicle 5.6 ± 0.96 vs bleomycin-senicapoc 2.39 ± 0.4, *p* = 0.0063; bleomycin-vehicle 6.44 ± 0.94, vs bleomycin-pirfenidone 6.57 ± 0.92, *p* = 0.92) (Figs. 5B and 6B). When we compare proliferation of alveolar capillary endothelial cells in bleomycin-injured segments of senicapoc and pirfenidone treated animals, there is significantly less proliferation in senicapoc-treated animals (bleomycin-senicapoc 2.39 ± 0.4 vs bleomycin-pirfenidone 6.57 ± 0.92, p = 0.0006).

## Discussion

Aberrant vascular remodelling has been implicated in the development and progression of pulmonary fibrosis. In this study, we have described the presence vascular remodelling during development of bleomycin-induced pulmonary fibrosis in our large animal model. We have demonstrated increased angiogenesis in the fibrotic lung segments which was attenuated by targeted blockade of K_Ca_3.1 ion channels. We found that VEGF expression, as well as alveolar capillary endothelial cells proliferation, was increased in bleomycin infused lung segments. Inhibition of the K_Ca_3.1 ion channel, but not pirfenidone treatment, suppressed VEGF expression and inhibited the proliferation of endothelial cells resulting in the attenuation of angiogenesis.

In the current study, the vascular density was significantly higher in bleomycin infused lung segments compared to saline controls, indicating that the stimulation of angiogenesis is one of the characteristic features in this model of pulmonary fibrosis. Very early studies implicated vascular remodelling in the pathogenesis of pulmonary fibrosis where they demonstrated neovascularization leading to anastomosis between pulmonary and systemic microvasculatures in the areas of fibrosis^2^. Others have shown that level of neovascularization is dependent on the regional pathology of the lung. Typically, a heterogeneous pattern of vascular remodelling is observed where there is increased density of CD34-positive alveolar capillaries in areas adjacent to extensive fibrosis, and a marked reduction of blood vessels inside the areas of extensive fibrosis in the lungs of IPF patients^7^.

In our sheep model, we found an increase in CD34-positive capillaries in fibrotic areas compared with healthy lung controls. While it has been shown that increased pulmonary vascular volume strongly predicts mortality in IPF patients^20^, we did not find a correlation between vascular density in fibrotic areas and poorer lung function. Another characteristic of the sheep model is the irregularly enlarged and dilated capillaries in parenchymal lung areas that exhibited fibrosis. The neo-formed abnormal blood vessel phenotype has been previously demonstrated in both animal models and IPF lungs^7,21^.

We found that there was a significant increase in the number of proliferating pulmonary capillary endothelial cells in bleomycin-infused lung segments, when compared to saline-infused control segments in the same sheep. Emerging evidence has shown a role for aberrantly activated pulmonary capillary endothelial cells in the progression of fibrosis. Moreover, a recent article has demonstrated a pivotal role of a hematopoietic-vascular niche, where aberrantly activated endothelial cells, perivascular macrophages and perivascular fibroblasts interact, in regulating alveolar repair and lung fibrosis. In that study involving mice with repetitive lung injuries, targeting the pulmonary capillary vascular niche by interfering with Notch signalling in pulmonary capillary endothelial cells promotes lung alveolar repair and ameliorates fibrosis^22^. Endothelial cells may contribute in many ways to the development of tissue fibrosis. Activated endothelial cells have been shown to secrete profibrotic cytokines such as TGF-β, connective tissue growth factor/CCN family member 2, and plasminogen activator inhibitor–1. Importantly, these profibrotic mediators directly recruit and activate fibroblasts to produce collagen which contribute to the pathology in the affected areas.

After confirming the vascular changes in response to bleomycin, we aimed to investigate the effects of different anti-fibrotic treatments on this process. We found that during the early fibrogenic response to lung injury, administration of the K_Ca_3.1 channel inhibitor, senicapoc, significantly improved vascular remodelling induced by bleomycin administration. It has been previously reported that K_Ca_3.1 channel promotes mitogenesis in several cell types including endothelial cells and inhibition of this channel via daily administration of TRAM-34, another selective K_Ca_3.1 channel inhibitor, significantly reduced VEGF and basic fibroblast growth factor-induced endothelial cell proliferation and angiogenesis *in vivo*^18^. The involvement of the K_Ca_3.1 ion channel in IPF has been demonstrated in several studies, as well as bleomycin-induced fibrosis^16,23–25^. Increased expression and activity of K_Ca_3.1 channels is found in IPF-derived human lung myofibroblasts, and inhibition of the channel through selective inhibitors attenuates pro-fibrotic behaviour in these cells, including proliferation, differentiation, wound healing, collagen secretion, contractility and α-smooth muscle actin expression in response to TGF-β1^24–26^. Inhibition of the K_Ca_3.1 ion channel attenuates established pulmonary fibrosis in the sheep model of pulmonary fibrosis^16^ and fibrosis in an *ex vivo* model of human lung fibrogenesis^26^. Whilst the expression and activity of K_Ca_3.1 has not been directly assessed in the endothelial cells from IPF lungs, we found that the lung injury-induced increase in the number of proliferating endothelial cells was significantly inhibited in sheep treated with senicapoc. The results from the current study suggest that the K_Ca_3.1 ion channel plays an important role in the neovascularization process in this animal model pulmonary fibrosis. Direct treatment of endothelial cells with bleomycin has been shown to induce production of profibrotic mediators *in vitro*^27,28^. In addition, this effect of bleomycin on endothelial cells has also been shown *in vivo*^29^. Moreover, endothelial cells may contribute to the development of fibrosis through endothelial-to-mesenchymal transition which is activated during fibrogenesis, and thus may serve as additional source of fibroblast-like cells that contribute to the fibrotic pathology^28,30^. The antifibrotic effect of senicapoc may thus be partly due to inhibition of endothelial cells proliferation and improving vascular remodelling. Overall, one of the attractive features of blockading the K_Ca_3.1 ion channel is that it targets multiple cell types and pathways involved in pulmonary fibrogenesis.

Pirfenidone is an approved anti-fibrotic for IPF, however, the mechanisms of action for this drug are still poorly characterised. In animal models, pirfenidone treatment modulated expression of numerous growth factors and cytokines that are shown to be relevant to the development of fibrosis, such as TGF-β, PDGF, SDF-1α/CXCL12, and TNF-α^31^. *In vitro* studies also support these findings and demonstrate that pirfenidone reduces fibroblast proliferation and modulates extracellular matrix deposition^31^. This study is the first to report the effect of pirfenidone on vascular remodelling associated with bleomycin-induced pulmonary fibrosis. In this study, pirfenidone has shown anti-angiogenic activity in blood vessels immuno-stained by collagen type IV antibodies, whereas CD34 stained capillaries were not significantly affected by pirfenidone. Collagen IV is a component of the basement membrane which is formed during maturation of capillary-like tubes thus it stains larger and more mature blood vessels^32,33^. In contrast, nascent microvessels are not collagen IV positive^33^. These small newly formed vessels are known to express CD34 on the cell surface^34^, while endothelial cells of larger veins and venules in massive fibrosis areas have been reported to be CD34 negative^7,34^. The difference effects of pirfenidone on collagen IV and CD34 stained blood vessels in this study suggests that pirfenidone may only exert an effect on larger and mature blood vessels, and not neovascularization. The data from the pirfenidone study group is in contrast with the senicapoc study cohorts in which K_Ca_3.1 blockade significantly attenuated CD34 stained nascent vessels.

There is augmented expression of VEGF in endothelial cells and alveolar type II epithelial cells of the highly vascularised alveolar septa in relatively preserved areas of the IPF lung^7^. The importance of VEGF to the disease process has been shown in a rat model of pulmonary fibrosis, where inhibition of the VEGF/VEGFR pathway with the angiogenesis inhibitor endostatin protects against vascular remodelling and fibrogenesis^9^. Expression of VEGF is increased in alveolar epithelial cells and airway epithelial cells in the lung segments exposed to bleomycin in this sheep model^14^. In the current study, we found that treatment with senicapoc, but not pirfenidone, was able to suppress bleomycin-induced VEGF expression in the lung. VEGF induces endothelial cell proliferation and migration, which gives rise to new blood vessels formation. This process of endothelial cell proliferation after stimulation by VEGF is initiated by a rise in intracellular calcium mediated through calcium-influx channels^35^. By maintaining the membrane potential negative, potassium channels promote this calcium entry and thus play an important role in regulating cell cycle progression^36^. It is plausible that reduced neovascularization observed in bleomycin-injured lungs in response to senicapoc treatment may be at least in part due to suppression of VEGF expression, as a result of K_Ca_3.1 channel blockade.

While the segmental challenge model has some inherent advantages, such as having internal control lung segments for comparing responses to those in the experimentally treated lung segments in the same sheep, there are some limitations with this protocol. One limitation is that systemic treatment with an individual drug results in that drug going to both the bleomycin- and saline-infused lung segments of the same sheep. One example outcome of the systemic treatment in our study is that vascular density scores were interestingly lower in the saline senicapoc segments when compared to the saline vehicle segments. As we are confident of our blinding protocols and analyses performed, we can only surmise that systemic senicapoc treatment via the oral route has a small effect on the saline-infused lung segment, as well as its obvious effect on the bleomycin-infused lung segment in the contralateral lung lobe. While this is an interesting observation, it is well out of the scope of the study to look at the mechanism operating here. Another limitation was that we did not measure the uptake of bleomycin into each of the bleomycin-infused lung segments. Nevertheless, this study, as well as our previous studies^14–16^, showing significant differences in data sets between the bleomycin- and saline-infused lung segments support the notion that bleomycin uptake overall is likely to be similar between the comparison groups. The differences we see between control and bleomycin-infused segments^14–16^ also support the contention that we can direct bleomycin repeatedly into precisely the same lung segment, and that the bleomycin doesn’t leach into other lung segments.

## Conclusions

Our observations indicate the presence of vascular remodelling in the parenchyma of bleomycin-induced fibrotic lungs in our large animal model. We found that senicapoc was more effective in reducing vascular remodelling than the currently approved drug pirfenidone. Senicapoc attenuated vascular remodelling through inhibiting endothelial cell proliferation and VEGF expression. These findings suggest a potential new mode of action for the novel drug senicapoc which may contribute to its efficacy in combatting pulmonary fibrosis.

## Methods

### Large animal model of pulmonary fibrosis

**Figure 7:**
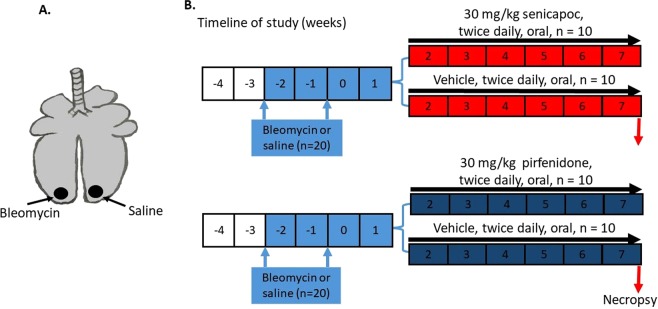
Induction of pulmonary fibrosis and therapeutic intervention in sheep. (**A**) Schematic diagram depicting the bleomycin and saline treatments administered to individual caudal lung segments in sheep. One lung segment received bleomycin, while the contralateral segment in the same sheep received saline as internal control. (**B**) Diagram showing the timeline for treatment of tested drugs (senicapoc or pirfenidone) or vehicle (methylcellulose), and euthanasia of experimental animals.

This study contains all new data derived from animals used in our previous study^16^ (senicapoc group), as well as new group of animals (pirfenidone group). Briefly, a total of 40 female Merino sheep, aged approximately one year, were used for this study. All experimental procedures performed on the sheep were approved by the Animal Experimentation Ethics Committee of the University of Melbourne (Parkville, VIC, Australia), which adheres to the Australian Code of Practice for the care and use of laboratory animals for scientific purposes. Fibrosis was induced in the caudal segments of lung of all treated sheep, as illustrated in Fig. 7A, using 3IU pharmaceutical grade bleomycin sulphate (Bleomycin; Hospira, Melbourne, VIC, Australia) at a concentration of 0.6 IU bleomycin/ml saline, with two doses, two weeks apart as previously described^16^. Sheep were euthanised at seven weeks after bleomycin injury by an intravenous overdose of barbiturate (Lethabarb, Virbac Animal Health, Australia). When we are sampling lung tissues at autopsy, the appropriate lung segment was removed from the whole lung, and then the airway which leads to the medial segment of the caudal region of the left or right lung (which we know has been infused with either bleomycin or saline) was identified. This lung segment was inflated with a mixture of 50% OCT/saline. Tissues were only sampled from these inflated lung segments. The tissues were stored in the freezer at −80 °C until required for further analysis.

### Administration of senicapoc and pirfenidone

We performed two separate studies, i.e. senicapoc and pirfenidone at separate times. For each study, sheep were randomly assigned into two groups each containing ten sheep (a total of 40 sheep for both studies). Two weeks after the second bleomycin dose, the sheep were given a five weeks regimen of twice daily oral administration of either; 30 mg/kg senicapoc (ICA- 17073; Icagen Inc., Durham, NC), a selective inhibitor of K_Ca_3.1 channel (n = 10 sheep)^16^; 30 mg/kg pirfenidone (n = 10); or vehicle control for both drugs (0.5% methylcellulose, Sigma-Aldrich, Castle Hill, NSW, Australia, 2x n = 10) (Fig. 7B). The solutions for pirfenidone and senicapoc was prepared in vehicle solution (0.5% methylcellulose in distilled water).

### Immunohistochemistry

Immunohistochemistry was performed on frozen sections of sheep lung tissue using previously described methods^16^. Primary antibodies and optimal dilutions used in this study were: anti-human collagen type IV (mouse monoclonal, 1:50, Dako, Kingsgrove, Australia); anti-CD34 (rabbit monoclonal, 1:50, Abcam, Waterloo, Australia); anti-human VEGF (A-20) (rabbit polyclonal, 1:50, Abcam). Secondary antibodies and optimal dilutions used were: anti-mouse rabbit IgG (HRP) (ab6728), 1:100 and anti-rabbit goat IgG/HRP, 1:100 (both from Abcam). for 1 hr at RT. Negative controls were included during each immunohistochemistry run by omission of primary antibody. The sections were counter stained with Mayer’s Haematoxylin solution. Antigen-antibody complex was visualised with 3,3′-diaminobenzidine.

### Quantitative evaluation of vascularity

Quantitative evaluation of vascularity was performed in the parenchyma of differentially treated lung tissue sections immuno-stained with type IV collagen and CD34 antibodies. For each differentially treated lung tissue sections, ten images were captured from fibroproliferative areas at x400 magnification using a digital camera (Leica ICC50 W, Leica Microsystems, Wetzlar, Germany) linked to a light microscope (Leica DM 500, Leica Microsystems, Wetzlar, Germany) and computer. Large blood vessels and bronchi were not included during image capturing. Images were analysed using Image-Pro® Plus software by H.B.D who was blinded to the treatment groups. Vascular density was determined by the percentage of total area surrounded by collagen type IV and CD34 positive endothelial cells per total area of parenchyma in the field of view. The value of vascular density for each of the ten fields was then averaged for each slide.

### Double immunofluorescence

Double immunofluorescence for CD34 (endothelial marker)/Ki-67 (proliferation marker) was performed on frozen lung tissue sections to examine proliferation of alveolar capillary endothelial cells. Primary antibodies and optimal dilutions used were: anti-CD34 (rabbit monoclonal, 1:50, Abcam) and rabbit anti-CD34 and anti-human Ki-67 (mouse monoclonal 1:75, Dako). The secondary antibodies were Alexa Fluor 488 anti-mouse goat IgG (1:100; Abcam) and Alexa Fluor 594 anti-rabbit goat IgG (1:100; Abcam). The coverslip was then mounted with glycerol mounting medium fortified with DAPI and DABCO™ (ab188804, Abcam). The slides were stored in the dark at 4 °C until viewed by Nikon A1R confocal microscopy. Immunofluorescence photomicrographs were captured with camera and NIS-Elements software. Proliferating capillary endothelial cells (cells that are positive for both labels) were counted from ten fields of highest endothelial proliferation areas for all differentially treated slides.

### Masson’s trichrome staining

Frozen sheep lung sections were stained with Masson’s trichrome using the trichrome stain kit (Sigma-Aldrich), according to instruction of the kit.

### Evaluation of fibrotic fraction

To quantify the degree of fibrosis or collagen content within the parenchymal tissue, we used a previously described method performed in our laboratory^15,16^. Briefly, Masson’s trichrome stained slide images were captured using a camera attached to microscope and computer. Ten fields from fibrotic areas excluding large blood vessels and bronchi were captured under x200 magnification. The images were analysed using Image-Pro® Plus software using the ‘colour selector’ tool to measure the area of collagen (blue stained tissue) within each field of view. The values for each of the ten images were averaged for each slide. The fibrosis fraction is expressed as a percentage per total field area.

### Statistical analysis

Statistical analysis was performed using GraphPad Prism for Windows Version 5.01 (GraphPad Software Inc., La Jolla, CA). Each parameter was assessed for Gaussian distribution using the Pearson omnibus and D’Agostino normality test. For data that are distributed normally, paired two-tailed *t* tests were performed to compare between treated and control segments within the same sheep whereas Wilcoxon matched-pairs signed rank test was performed for data that did not meet assumptions for Gaussian distribution. Differences between the drug (senicapoc or pirfenidone) treated sheep and vehicle (methylcellulose) treated sheep (between lobes of different animals) were assessed using a Student’s *t* test for data with a normal distribution and a Mann-Whitney test for data with a nonparametric distribution. Correlation analysis were performed using correlation coefficient (*r*). A *p* value less than 0.05 was defined as significant difference. All values are reported as means ± SEM.

## Data Availability

The datasets generated during and/or analysed during the current study are available from the corresponding author on reasonable request.
